# *Hermetia illucens* chitosan: indirect and direct antimicrobial activity of an innovative biopolymer for clinical and pharmaceutical applications

**DOI:** 10.1007/s00253-025-13643-7

**Published:** 2025-12-19

**Authors:** Guarnieri Anna, Fusco Alessandra, Scieuzo Carmen, Salvia Rosanna, Donnarumma Giovanna, Falabella Patrizia

**Affiliations:** 1 Department of Basic and Applied Sciences, University of Basilicata, Via Dell’Ateneo Lucano 10, 85100 Potenza, Italy; 2 Department of Life Sciences, Health and Health Professions, Link Campus University, 00165 Rome, Italy; 3 Department of Experimental Medicine, University of Campania “Luigi Vanvitelli”, 80138 Naples, Italy; 4 Spinoff XFlies S.R.L, University of Basilicata, Via Dell’Ateneo Lucano 10, 85100 Potenza, Italy

**Keywords:** *Hermetia illucens*, Chitosan, Indirect and direct antimicrobial activity, Pharmaceutical field

## Abstract

**Abstract:**

The increasing spread of antimicrobial resistance has prompted the search for innovative alternatives to conventional antibiotics. Chitosan, a biopolymer derived from chitin, is known for its broad-spectrum antimicrobial activity. This study evaluated both direct and indirect antimicrobial activity of chitosan obtained from *Hermetia illucens*, a novel and sustainable source compared to the traditionally crustacean-derived biopolymer. Chitosan produced from *H. illucens* larvae, pupal exuviae and adults, through heterogeneous and homogeneous deacetylation, was tested for both its indirect and direct antimicrobial effects. The indirect effect was evaluated by measuring the induction of Human Beta-Defensin-2 (*HBD-2*) expression in HaCaT keratinocytes stimulated with lipopolysaccharide of *Salmonella typhimurium*, a Gram-negative bacterium. The direct antimicrobial activity was assessed against Gram-positive pathogens (*Enterococcus faecalis*, *Staphylococcus epidermidis*, and *Streptococcus agalactiae*), using a microdilution assay and plate colony count. Results demonstrated significant bacteriostatic effects at 0.5 mg/mL, with some samples, particularly the homogeneous unbleached pupal exuviae chitosan and the heterogeneous unbleached larvae chitosan, comparable to or even superior to commercial chitosan in terms of biological activity. Furthermore, insect-chitosan significantly up-regulated *HBD-2* expression, suggesting immunomodulatory activity. These findings validated *H. illucens* as a promising alternative source of chitosan with dual antimicrobial activity, and supported its potential use in clinical, pharmaceutical and biomedical applications.

**Key points:**

• *Insect-chitosan activates innate immunity via strong HBD-2 induction*

• *Chitosan samples showed notable growth-inhibition toward key Gram-positive strains*

• *Hermetia illucens chitosans provide efficacy comparable or superior to the commercial biopolymer*

## Introduction

Antimicrobial resistance (AMR) has become one of the major global challenges of the twenty-first century, due to the rapid increase in AMR-associated infections and the scarcity of new antimicrobial drugs developed to counter the problem (Llor and Bjerrum 2014; Prestinaci et al. 2015).

Developing new antibiotics is currently highly challenging in several aspects, both technical and economic. Since 1980, the number of antibiotics approved by the *Food and Drug Administration* has been reduced from 20 to 6%. This is due to the development of new antimicrobial agents being economically burdensome and it is extremely difficult for pharmaceutical companies to make a profit, thus making it considerably tricky to release them onto the market, despite the urgent need for new drugs (Gargate et al. 2025). This need is mainly linked to the fact that pathogens have become increasingly resistant to antibacterial molecules, but the discovery of antibiotics has not kept in line with this process (Laxminarayan et al. 2013). Furthermore, the release of antibiotics into the environment by industrial manufacturers can cause contamination of water and the environment, (Larsson 2014; Larsson et al. 2007) leading to increased antibiotic resistance, particularly in areas affected by large-scale industrial contamination (Li et al. 2009; Johnning et al. 2013). In general, new antimicrobial discovery can help significantly reduce the period of hospitalization and healthcare costs associated with infections caused by resistant pathogens (Dixon and Duncan 2014), promoting innovation through the use of advanced technologies. For all these reasons, in order to counteract pathogenic infections, research efforts are focused on identifying new natural molecules with antibacterial activity. including the potential use of chitosan, a biopolymer obtained from chitin deacetylation (Croisier and Jérôme 2013). Chitosan has numerous advantages from a biological point of view and insect chitosan also in terms of economics and sustainability. Thanks to its stable chemical structure and non-toxic properties, chitosan is biocompatible with different organs, tissues and cells (Yadav et al. 2024). Furthermore, chitosan is highly susceptible to hydrolytic enzymatic degradation in the human body, mainly by lysozymes (Roman et al. 2020). At the same time, it is a polysaccharide with broad-spectrum antimicrobial properties against several pathogenic microorganisms (Pal et al. 2021), including both Gram-negative and Gram-positive bacteria (Yoshida et al. 2021). Chitosan antimicrobial activity relies on several mechanisms. In acidic environments, the biopolymer becomes protonated via its -NH_3_⁺ cationic groups, which interact with bacterial membranes, increasing their permeability and causing the release of intracellular contents (Tantala et al. 2021; Moradi et al. 2023). Chitosan can also interfere with protein and mRNA synthesis (Kuo et al. 2006) and hinder enzyme activity, fostering toxin production (Ardean et al. 2021). Furthermore, the biopolymer could also achieve antibacterial activity through the formation of a multi-polymer membrane that would limit nutrient availability to the bacterial cell (Ardean et al. 2021; Costa et al. 2012). Therefore, the biopolymer may represent a viable and cost-effective alternative in cases of antibiotic resistance (Croisier and Jérôme 2013). This cost-effectiveness is even morepronounced when chitosan is obtained from an alternative source rather than crustaceans, which are the commercially used source at the industrial scale (Triunfo et al. 2022). Ready availability, easy breeding conditions, and resistance to pathogens make insects a viable alternative source of chitin and chitosan (Hillyer 2016; Vallet-Gely et al. 2008).

Currently, one of the most interesting is *Hermetia illucens*, a widespread bioconverting dipteran, reared in most part of European insect farms, able to produce raw materials, rich in different bioactive molecules, including chitin (Derrien and Boccuni 2018; Jucker et al. 2020; Scala et al. 2020; Triunfo et al. 2021; Franco et al. 2021a, b, 2022; Scieuzo et al. 2022, 2023). The insect’s peculiarity lies in the way that chitin, and thus chitosan, can be produced by heterogeneous and homogeneous deacetylation (Triunfo et al. 2022, 2024) from waste products of the breeding itself (adults and pupal exuviae) and from larvae, with chemical, physical, and biological properties comparable to those of crustacean chitosan (Guarnieri et al. 2022, 2025; Coltelli et al. 2025; Ianniciello et al. 2025; Giani et al. 2025; Marsico et al. 2025a, b). Previous studies demonstrated the antibacterial properties of *H. illucens*-derived chitosan (Guarnieri et al. 2022) as well as its immunomodulatory properties (Fusco et al. 2025).

In case of infection, the presence of microorganisms has an effect on human innate immunity; they are able to induce the expression of cationic peptides, belonging to the defensin family, which contribute to broad-spectrum innate immunity, and that act by damaging bacterial cell membranes (Raj and Dentino 2002; Ganz 2003; Bulet et al. 2004). Defensins (α and β) belong to a broader class of host defence peptides, also known as HDPs (Mygind et al. 2005; Sahl et al. 2005; Crovella et al. 2005; Dhople et al. 2006), and they positively influence the immune system by modifying host gene expression, limiting the production of pro-inflammatory cytokines triggered by lipopolysaccharide (LPS). Peptide production can be induced by bacterial LPS and other inflammatory stimuli (Diamond et al. 1996; Zhang et al. 2000). These peptides also have the additional capacity to bind LPS, thus indirectly counteracting the cellular signalling mechanisms activated through LPS, in order to avoid excessive inflammatory responses that could lead to critical conditions such as sepsis (Hancock 2001). According to some studies, the fundamental microbiological difference between α-defensins and β-defensins lies primarily within the framework that the α-defensins act on a wide variety of bacteria, both Gram-negative and Gram-positive, a large number of fungi and sometimes even viruses, whereas β-defensins have a smaller spectrum, acting mainly on Gram-negative bacteria and fungi (Donnarumma et al. 2015). Human Beta Defensin-2 (*HBD*−2) is an inducible antimicrobial peptide, that can be found in different tissues, including the outermost layer of the epidermis (Harder et al. 1997), oral cavity epithelium (Dale and Krisanaprakornkit 2001), corneal epithelium (McDermott et al. 2003) and intestinal epithelia. Several *in vitro* studies demonstrated *HBD*−2 antibacterial activity against yeasts and bacteria (Bals et al. 1998; Valore et al. 1998).

 Danti et al*.* (2019) carried out *in vitro* tests to verify the efficacy and the safety of complexes of chitin (functionalised with lignin, a natural origin polymer) on human keratinocytes, and they proved the good anti-inflammatory capacity of these innovative complexes, as well as their ability to stimulate the *HBD*−2 expression. This finding provides important evidence supporting the indirect antimicrobial activity of the biopolymer. For all these reasons, the study of biodegradable and biocompatible natural molecules, such as chitosan, opens up new perspectives in personal care and human health. Furthermore, a biopolymer with both indirect and direct antimicrobial activity, especially against common human pathogens, could help address the critical clinical issue of the widespread emergence of antimicrobial-resistant Gram-positive strains in recent decades (Song 2011).

Particularly, enterococci and streptococci are Gram-positive bacteria that play an important role in human diseases (Calatrava 2002). These two bacterial strains were originally classified in the same genus, but are now recognised as taxonomically distinct (Hardie and Whiley 1997).

Enterococci are able to survive in stressful and hostile environments (Mancuso et al. 2021). They are commensals normally found in humans, adapted to nutrient-rich and oxygen-poor environments (for example, the oral cavity and gastrointestinal tract) (Jett et al. 1994).

Although more than 50 species of enterococci have been identified, *Enterococcus faecalis* is considered the most pathogenic species, responsible for nosocomial infections, including catheter-associated urinary infections, endocarditis and bacteremia in immunocompromised individuals (García-Solanche and Rice 2019).

Streptococci are a heterogeneous group comprising a variety of species capable of causing different diseases, ranging from minor morbid tissue infections to life-threatening sepsis (Nitsche-Schmitz and Chhatwal 2013). Among the most important pathogens of this group, there is *Streptococcus agalactiae* (group B streptococcus–GBS) (Caliot et al. 2012).

*S. agalactiae* is a well-known agent of invasive infections in newborn children and pregnant women. Invasive neonatal infection is mainly due to maternal colonisation with GBS in the gastrointestinal or genitourinary tract (Tavares et al. 2022). It has now also become a relevant pathogen in non-pregnant adults, particularly in patients with pre-existing diseases (Tyrrell et al 2000).

Together with streptococci, staphylococci are among the main causes of bacterial and health infections worldwide (Stoneham et al. 2021). The widespread use of medical devices and the inappropriate or prolonged use of antibiotics have contributed to the recent rise of *Staphylococcus epidermidis* as a significant nosocomial pathogen (Saffari et al. 2016).

*S. epidermidis*, a Gram-positive and coagulase-negative bacterium, is among the main microorganisms in human skin and mucous membranes and can cause nosocomial infections, especially due to the widespread use of medical devices (von Eiff et al. 2002). This bacterium is able to reduce the permeability and penetration of antibiotics, thanks to its ability to form biofilms (Hall-Stoodley et al. 2004).

The aim of this work was to test, for the first time, both the indirect and direct antimicrobial activity of chitosan from *H. illucens.* Chitosan was obtained either by heterogeneous or homogeneous deacetylation. Its effects were evaluated on the *HBD*−2 peptide after stimulation with LPS of *Salmonella enterica* subsp*. enterica* serovar Typhimurium, a Gram-negative bacterium, and on Gram-positive pathogenic bacteria, namely *E. faecalis*, *S. epidermidis*, and *S. agalactiae*.

## Materials and methods

### *Hermetia illucens* rearing

As reported in Triunfo et al. 2022, 2024, insect biomasses were provided by Xflies s.r.l (Potenza, Italy). Specifically, following *H. illucens* eggs hatching, larvae were reared under controlled environmental conditions (27 ± 1 °C, 70% ± 5% relative humidity, and a 12 h light:12 h dark cycle) and fed on the standard Gainesville diet consisting of 50% wheat bran, 30% alfalfa, and 20% corn meal (Scieuzo et al. 2023; Hogsette 1992).

*H. illucens* larval stages are followed by pre-pupal and pupal stages which allow the adult to emerge. In this way, larvae, pupal exuviae and adults, at the end of the insect life cycle, are recovered in order to be processed for biopolymers extraction and production.

### Chitin extraction and chitosan production

Raw insects were oven-dried (Conlabo s.r.l., Potenza, Italy) and ground into powder (Waring Commercial Stamford, USA). Subsequently, the samples were subjected to chitin extraction by demineralisation and deproteinisation processes in order to obtain unbleached chitin, the first batch of samples tested. Another part of the unbleached chitin was subjected to a bleaching step, in order to obtain also the bleached biopolymer (Triunfo et al. 2022). Unbleached and bleached chitin were then oven-dried and subjected to both heterogeneous and homogeneous deacetylation (Triunfo et al. 2022, 2024). This last step allowed obtaining unbleached chitosan and bleached chitosan, both heterogeneously and homogeneously deacetylated, from all *H. illucens* biomasses.

### Chitosan indirect antimicrobial activity

#### Cell culture

As previously described in Fusco et al. 2025, HaCaT cells (Elabsciences) were cultured in Dulbecco’s Modified Eagle Medium (DMEM) (Gibco, Thermo Fisher Scientific, Waltham, MA, USA) supplemented with 10% fetal bovine serum (FBS) (Gibco, Thermo Fisher Scientific, Waltham, MA, USA), 1% penicillin–streptomycin (Gibco, Thermo Fisher Scientific, Waltham, MA, USA), and 1% l-glutamine (Gibco, Thermo Fisher Scientific, Waltham, MA, USA) and maintained in a humidified atmosphere with CO_2_, before being seeded and grown to approximately 80% confluence.

#### Evaluation of *H. illucens* chitosan-immunomodulating effects

Chitosan solutions from *H. illucens* larvae, pupal exuviae, and adults were prepared by dissolving the biopolymer (in both heterogeneously and homogeneously deacetylated forms) in 17 mM acetic acid (Sigma-Aldrich St. Louis, Missouri, USA), adjusted to pH ~ 7.0 and diluted in DMEM to reach a final concentration of 0.5 mg/mL for cell treatment (Fusco et al. 2025). Semi-confluent HaCaT cells were exposed to chitosan solutions for 24 h to assess their viability, while inflammation was induced using *S.* Typhimurium LPS (20 µg/mL), with or without chitosan for 6 and 24 h at 37 °C. Commercial chitosan (Sigma-Aldrich, St. Louis, Missouri, USA) was employed as a control.

After extracting mRNA, complementary DNA (cDNA) was used in qPCR to evaluate the expression levels of *HBD-2*. In Table 1, the primer sequences used for the qPCR are reported.

**Table 1:** Primers used in the qPCR experiments

Gene	Primer sequences	Conditions	Amplicon size (bp)
*HBD-2*	5′-GGATCCATGGGTATAGGCGATCCTGTTA −3	5″ at 95 °C, 6″ at 63 °C,	198
	5′-AAGCTTCTCTGATGAGGGAGCCCTTTCT-3′	10″ at 72 °C for 45 cycles	

#### Chitosan direct antimicrobial activity

*Sample preparation and microdilution assay*

Chitosan samples were prepared as described in the previous section.

The bacterial strains of *E. faecalis* (ATCC® 9027™) and *S. epidermidis* (ATCC® 35,984™) and a clinical isolate of *S. agalactiae* were grown in Brain Heart Infusion (BHI) broth (OXOID) at 37 °C overnight in aerobic conditions. The *S. agalactiae* clinical isolate was obtained by the Unity of Microbiology and Virology of the University of Campania “Luigi Vanvitelli”. Minimal inhibitory concentrations (MICs) of the samples were determined in a 96-well plate by broth microdilution assay, according to the European Committee on Antimicrobial Susceptibility Testing (EUCAST). Bacterial suspensions obtained as previously described were diluted to an Optical Density (O.D. _600_) around 0.3 (corresponding to 1 × 10^8^ CFU mL^−1^ approximatively^)^ and 1 µL of these dilutions was added to 200 µL of fresh BHI in each well, to reach a final concentration of 1 × 10^6^ CFU mL^−1^. Chitosan samples were added to the bacterial suspension in each well, with a final concentration ranging from 0.5 to 0.0015 mg/mL (in serial dilutions). Positive control wells were carried out to contain bacteria in BHI. Negative controls included the compounds diluted in BHI without bacteria. Medium turbidity was measured by a microtiter plate reader (Tecan, Milan, Italy) at 600 nm. Absorbance detected was proportional to bacterial growth.

Colony forming units (CFUs) counting for the determination of the minimum bactericidal concentration After performing spectrophotometer readings, the unique *H. illucens* chitosan concentration at which inhibition or reduction of *E. faecalis*, *S. epidermidis*, and *S. agalactiae* growth was observed (0.5 mg/mL) was subjected to CFUs counting.

The contents of the wells were serially diluted in phosphate buffered saline (PBS) and spotted in triplicate onto the agar plates. A positive control, consisting of bacteria without chitosan, was also included in each experiment. After 24 h of incubation at 37 °C, CFUs were counted. The average colony count from the triplicates of each sample was used to determine the bactericidal effect of chitosan from *H. illucens*. Plate colony counts were employed to determine the minimum bactericidal concentration (MBC).

*Colony Forming Units (CFUs) counting* *for the determination of the minimum bactericidal concentration*

 After performing spectrophotometer readings, the unique *H. illucens* chitosan concentration at which inhibition or reduction of *E. faecalis*, *S. epidermidis* and *S. agalactiae* growth was observed (0.5 mg/mL), were subjected to CFUs counting. The contents of the wells were serially diluted in PBS and spotted in triplicate onto the agar plates. A positive control, consisting of bacteria without chitosan, was also included in each experiment. After 24 hours of incubation at 37 °C, CFUs were counted. The average colony count from the triplicates of each sample was used to determine the bactericidal effect of chitosan from *H. illucens*. Plate colony counts were employed to determine the minimum bactericidal concentration (MBC). 

#### Statistical analysis

Experiments were carried out in triplicate and results were expressed as mean ± standard deviation. Data were analyzed with one-way and two-way ANOVA, with Bonferroni *post hoc* test. All statistical analyses were performed using GraphPad Prism version 6.01 for Windows (GraphPad Software, La Jolla, California USA—www.graphpad.com).

## Results

### Indirect antimicrobial activity

Experiments aimed at evaluating the effect of chitosan from *H. illucens* on HaCaT keratinocytes were carried out after having preliminarily performed cell viability studies of the biopolymer, as previously reported in Fusco et al. (2025).

**Fig. 1:**
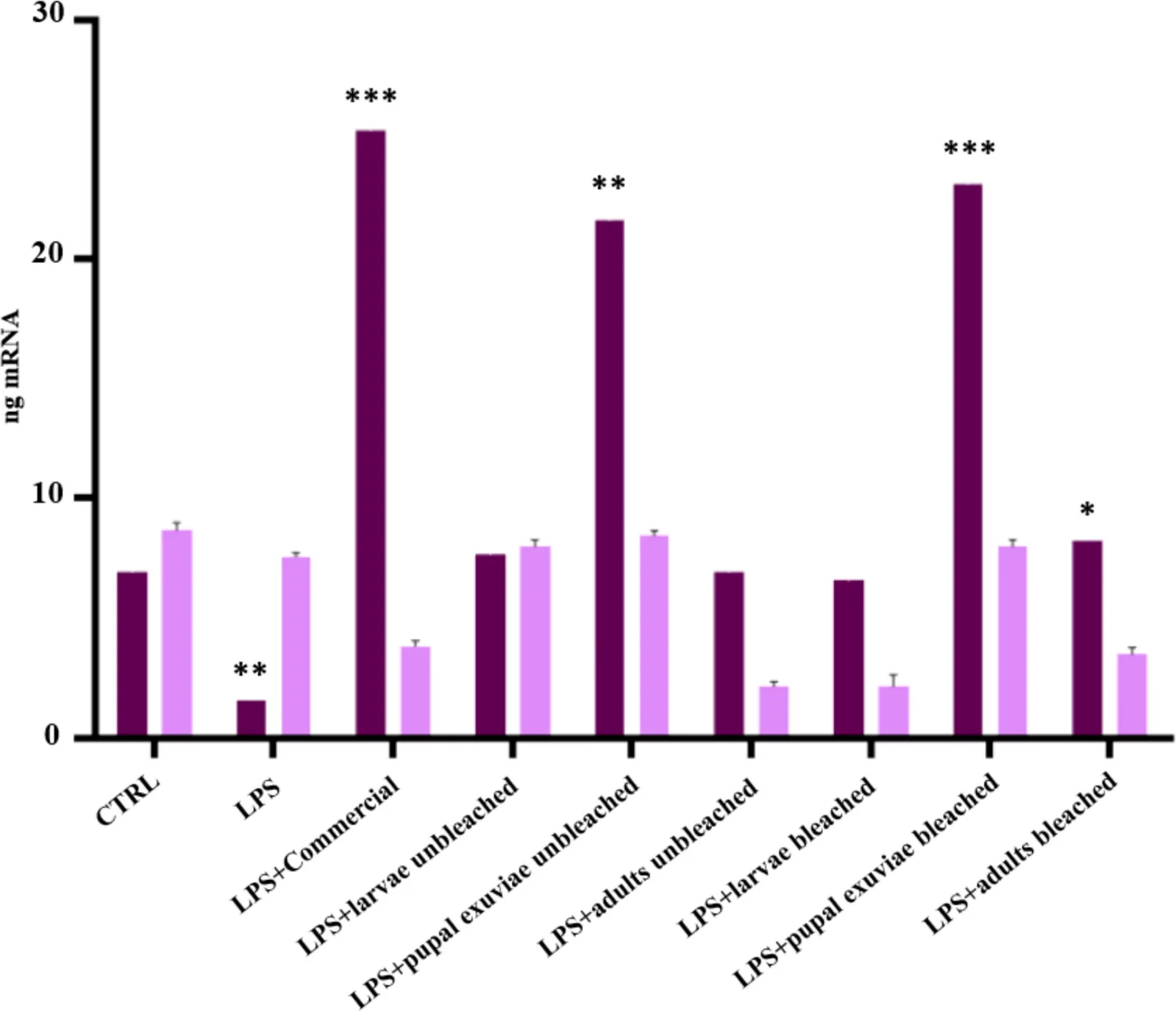
qPCR revealed the expression levels of *HBD-2* in HaCaT cells treated with LPS and heterogeneous chitosan from *H. illucens*, both bleached and unbleached, 6 h (dark purple bars) or 24 h (light purple bars) post treatment. Data are expressed as relative mRNA expression ± standard deviation in each group and are representative of three different experiments. Significant differences are indicated by *p < 0.05, **p <0.01, ***p <0.001. Data were analyzed with two-way ANOVA and Bonferroni *post hoc *test

**Fig. 2:**
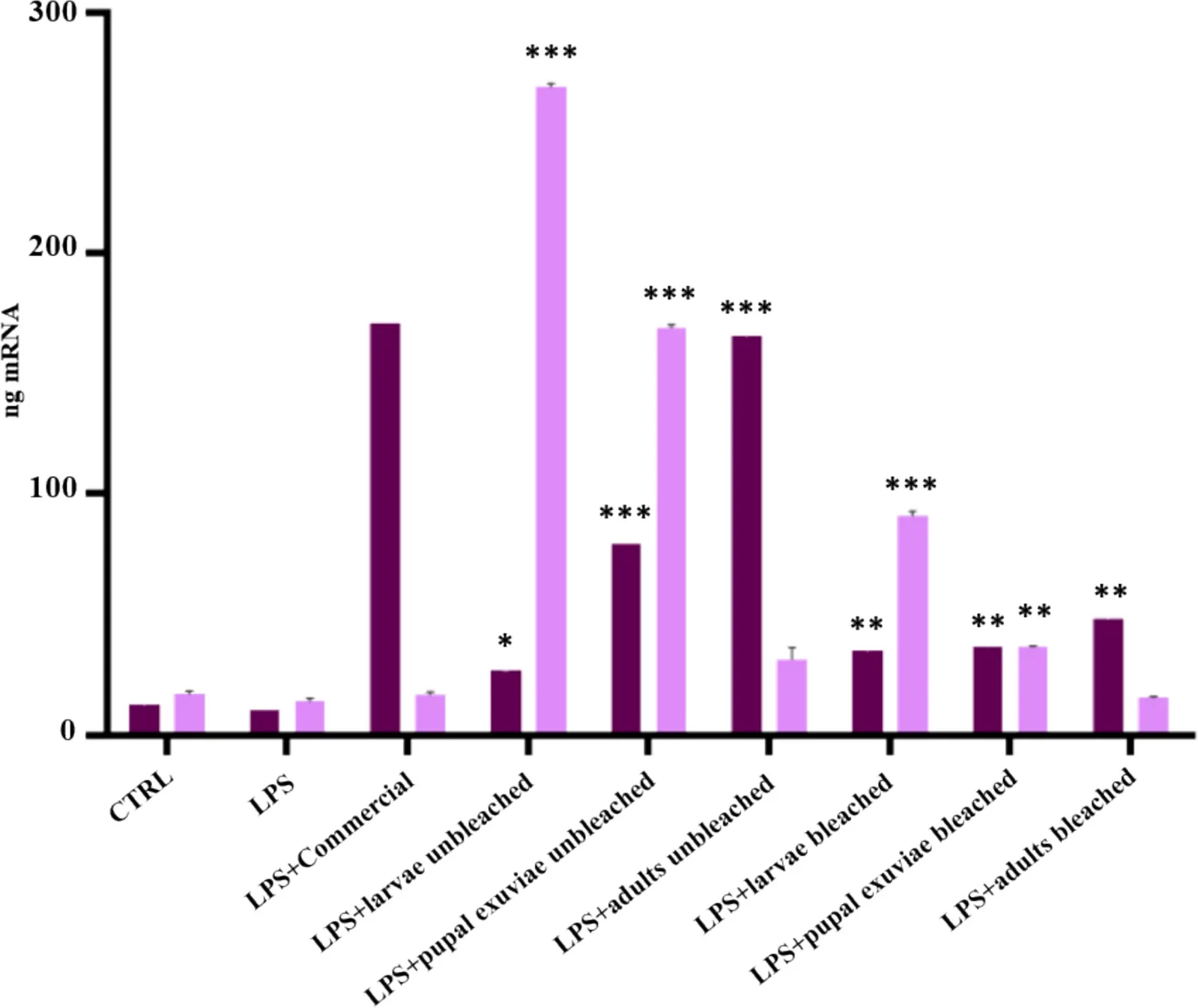
qPCR revealed the expression levels of *HBD-2* in HaCaT cells treated with LPS and homogeneous chitosan from *H. illucens*, both bleached and unbleached, 6 h (dark purple bars) or 24 h (light purple bars) post treatment. Data are expressed as relative mRNA expression ± standard deviation in each group and are representative of three different experiments. Significant differences are indicated by *p <0.05, **p <0.01, ***p <0.001. Data were analyzed with two-way ANOVA and Bonferroni *post hoc *test

The results obtained showed that all *H. illucens* chitosan samples tested had 97–100% cell viability. The capacity of *H. illucens* chitosan to induce the expression of the *HBD-2* gene, after 6 h and 24 h of treatment, was tested to confirm the hypothesis of its indirect antibacterial activity. Chitosan samples effectively upregulated the expression of *HBD-2* in HaCaT keratinocytes. Quantitative PCR analysis demonstrated a significant immunomodulatory effect of chitosan in counteracting the LPS-induced inflammatory response. Results obtained showed that, with regard to heterogeneous chitosan samples (Fig. 1), all insect-derived chitosan samples, except that from larvae, induced higher levels of *HBD-2* gene expression at 6 h after treatment. This induction was not achieved either by the control or by LPS treatment alone. The best peptide induction was obtained with chitosan derived from pupal exuviae. Particularly, heterogeneous chitosan derived from pupal exuviae proved to be the most effective, showing an efficacy comparable to that of commercial chitosan for the bleached sample and slightly lower for the unbleached one. At 6 h post-treatment, all homogeneous chitosan samples from *H. illucens*, both bleached and unbleached, modulated an enhancement of *HBD-2* gene expression levels on HaCaT cells. Notably, the greatest increase was gained from unbleached pupal exuviae and unbleached adult chitosan. At 24 h after LPS induction, on the other hand, unbleached pupal exuviae kept a good level of induction of peptide expression, although the greatest increase was obtained from the biopolymer from unbleached larvae. As shown in Fig. 2, after 24 h, the expression levels of *HBD-2* induced by the treatments with homogeneous chitosan from *H. illucens* are significantly higher than the expression induced by chitosan derived from crustaceans. This finding is particularly relevant as it validates the indirect antimicrobial activity of insect chitosan and demonstrates its higher efficacy compared to commercial chitosan, suggesting its potential as an effective substitute also because of this biological property.

### Direct antimicrobial activity

When conducting the microdilution assay experiments, it was found that only the highest concentration (0.5 mg/mL) showed an inhibitory effect on *E. faecalis*, *S. epidermidis*, and *S. agalactiae*, the tested Gram-positive bacteria. At lower concentrations, the absorbance values for all *H. illucens* chitosan samples and for the commercial biopolymer were always comparable to the control (data not shown).

**Fig. 3:**
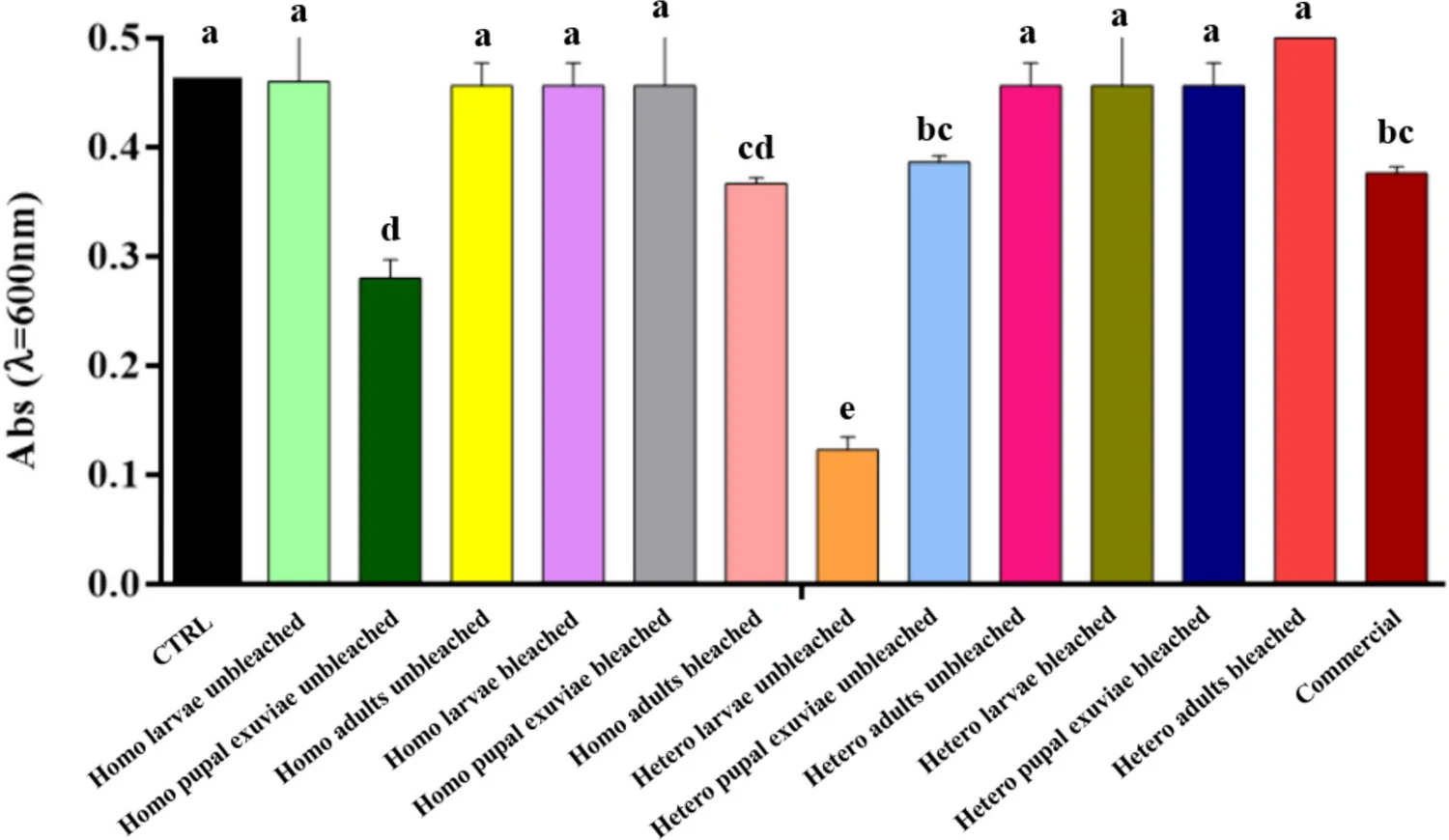
Results of microdilution assay of bleached and unbleached chitosan, both homogeneously and heterogeneously deacetylated, from larvae, pupal exuviae and adults of *H. illucens* at 0.5 mg/mL concentration on *E. faecalis*. The black bar indicates the bacterial culture control, while the red bar represents the commercial chitosan result. Different letters indicate significant differences (*p* < 0.05) between absorbance values of the bacterial culture alone and that of bacteria treated with each treatment. Data are analyzed with one-way ANOVA and Bonferroni* post hoc* test

Microdilution assay results demonstrated that the absorbance values were kept high for most of the samples obtained by homogeneous deacetylation (Fig. 3). Among them, the only decrease in absorbance values was observed in bleached adults and unbleached pupal exuviae samples, with the latter providing the best result.

Heterogeneous chitosan samples showed different behaviors depending on the treatment investigated. Chitosan from unbleached larvae represented the sample with the lowest absorbance level, which resulted in a statistically significant reduction and thus a greater inhibition of *E. faecalis* growth, in comparison to all the other samples employed in the experiment. In contrast, all bleached heterogeneous chitosan samples showed absorbance levels similar to the control. Treatment with commercial chitosan induced a statistically significant reduction compared to the control, but still higher than that caused by homogeneous chitosan from unbleached pupal exuviae and from the heterogeneous biopolymer from unbleached larvae.

**Fig. 4:**
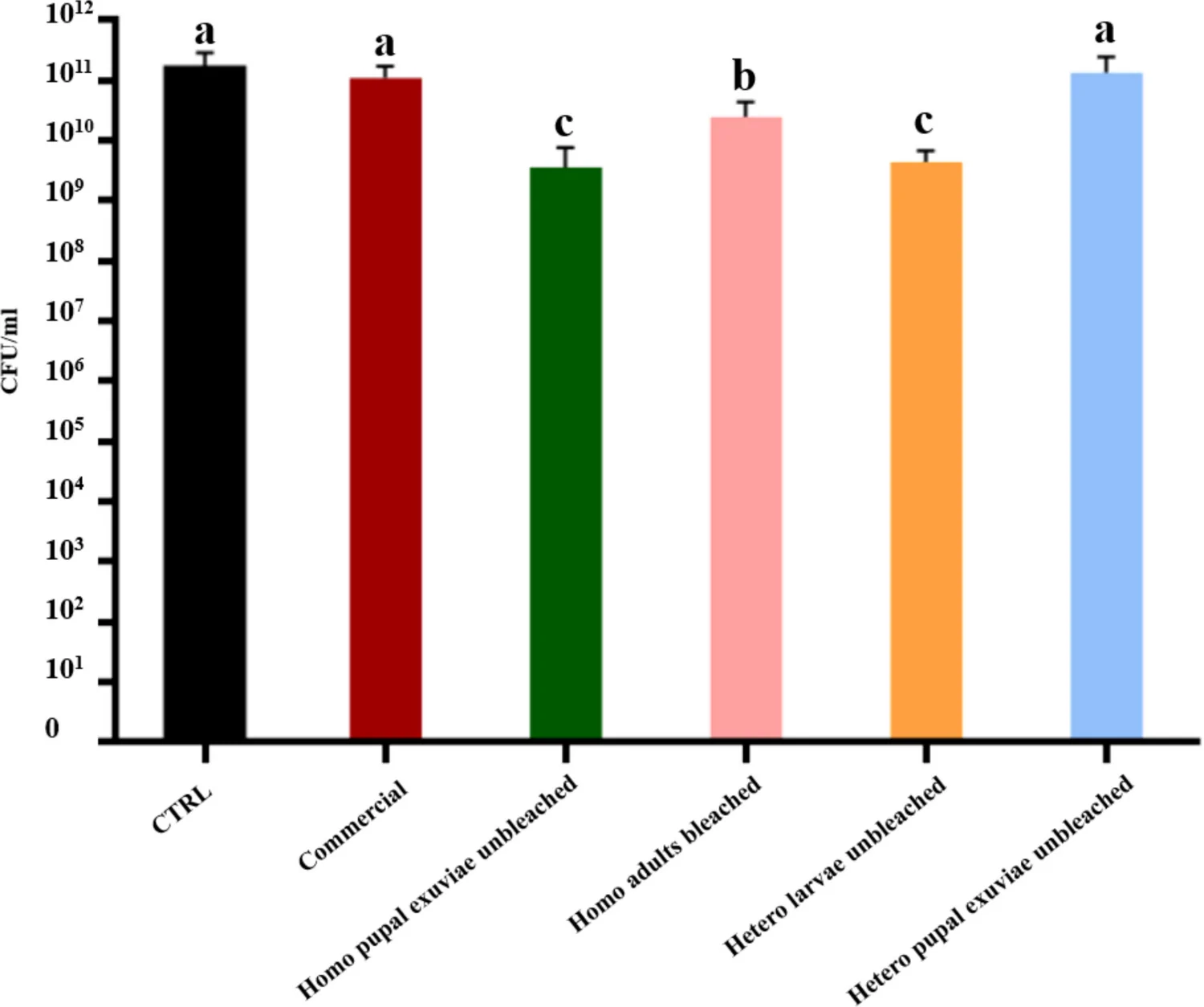
Results of CFUs counting of bleached and unbleached chitosan that gave MIC statistically significant values, both homogeneously and heterogeneously deacetylated, from larvae, pupal exuviae and adults of *H. illucens* at 0.5 mg/mL concentration on *E. faecalis*. The black bar indicates the bacterial culture control, while the red bar represents the commercial chitosan result. Different letters indicate significant differences (*p* < 0.05) between CFU/mL of the bacterial culture alone and that of bacteria treated with each treatment. Data are analyzed with one-way ANOVA and Bonferroni *post hoc* test

When considering the CFUs counting (Fig. 4) of unbleached homogeneous chitosan from pupal exuviae, the result (2 × 10⁹ CFUs/mL) was statistically significant compared to both the bacterial control and the commercial chitosan (both 1 × 10^11^ CFUs/mL); however, the absolute CFUs/mL count does not provide evidence of strong bactericidal activity for insect-derived chitosan from this biomass. Homogeneous bleached adults chitosan (2 × 10^1^⁰ CFUs/mL) and heterogeneous unbleached chitosan from larvae (4 × 10⁹ CFUs/mL) also resulted in a reduction compared to the controls, although the reduction in microbial load was modest and did not reach strong bactericidal thresholds. No significant difference in colony count was found for unbleached heterogeneous chitosan from pupal exuviae.

**Fig. 5:**
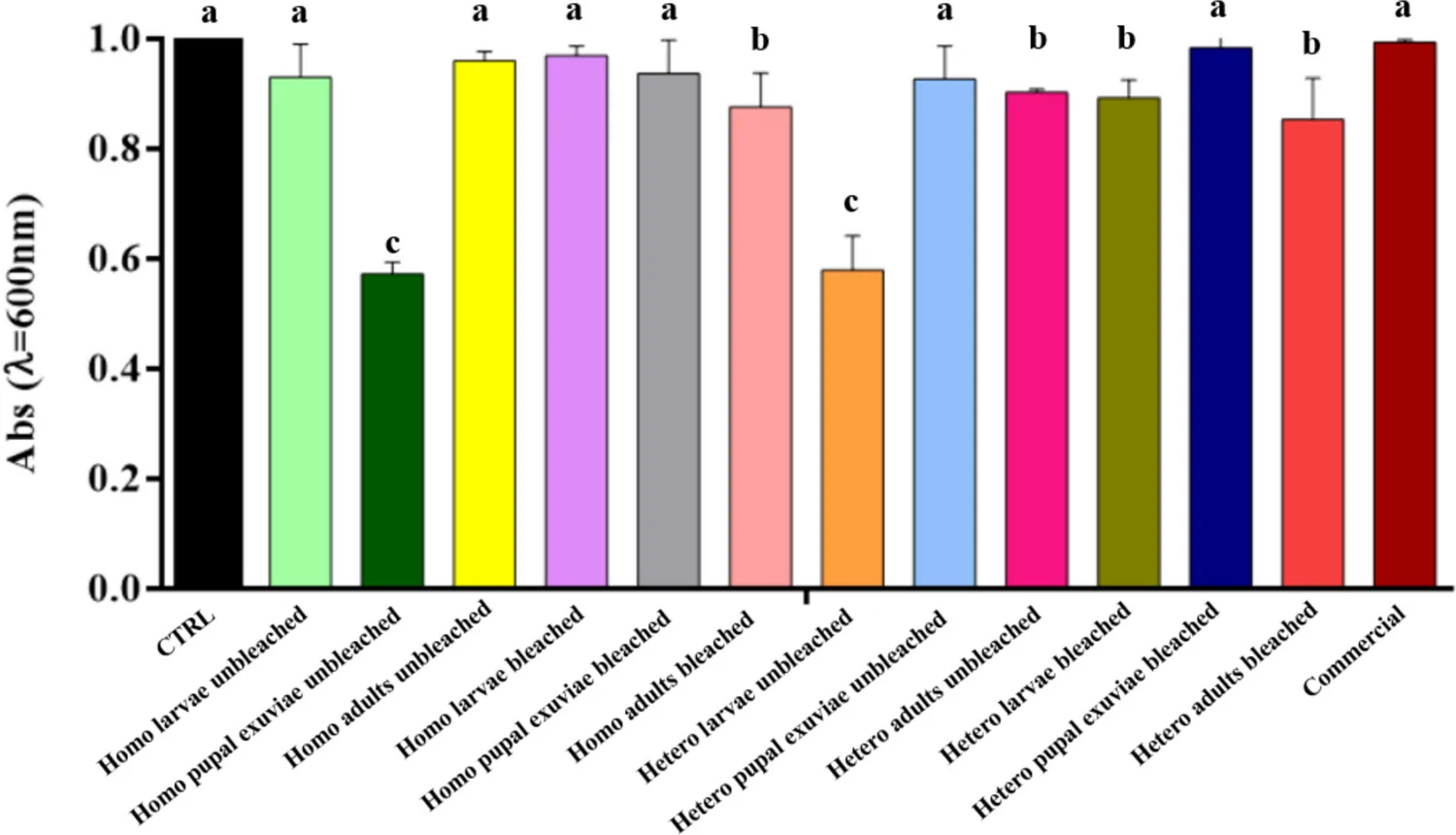
Results of microdilution assay of bleached and unbleached chitosan, both homogeneously and heterogeneously deacetylated, from larvae, pupal exuviae and adults of *H. illucens* at 0.5 mg/mL concentration on *S. epidermidis*. The black bar indicates the bacterial culture control, while the red bar represents the commercial chitosan result. Different letters indicate significant differences (*p* < 0.05) between absorbance values of the bacterial culture alone and that of bacteria treated with each treatment. Data are analyzed with one-way ANOVA and Bonferroni *post hoc* test

Analysis of the data obtained with chitosan samples treatment on *S. epidermidis* (Fig. 5) showed that treatments, particularly those of bleached chitosan from *H. illucens*, gave absorbance values comparable to those of the control and commercial chitosan, suggesting that these samples are not able to effectively counteract microbial growth. Notably, bleached chitosan samples, both heterogeneous and homogeneous, from larvae and pupal exuviae, showed high absorbance values, statistically indistinguishable from the control, with a slight decrease in absorbance only when treated with heterogeneous chitosan from bleached larvae and adults and homogeneous chitosan from adults.

On the contrary, homogeneous unbleached chitosan from pupal exuviae and heterogeneous unbleached chitosan from larvae showed a significant reduction in absorbance, yielding a statistically significant result when compared to both the bacterial culture control and commercial chitosan. Interestingly, within both the group of chitosan obtained through homogeneous deacetylation and the group obtained through heterogeneous deacetylation, the unbleached samples from each biomass were those that always showed comparable or lower absorbance levels than the respective bleached sample, apart from the homogeneous chitosan sample from adults, where the absorbance levels were lower for the bleached sample.

**Fig. 6:**
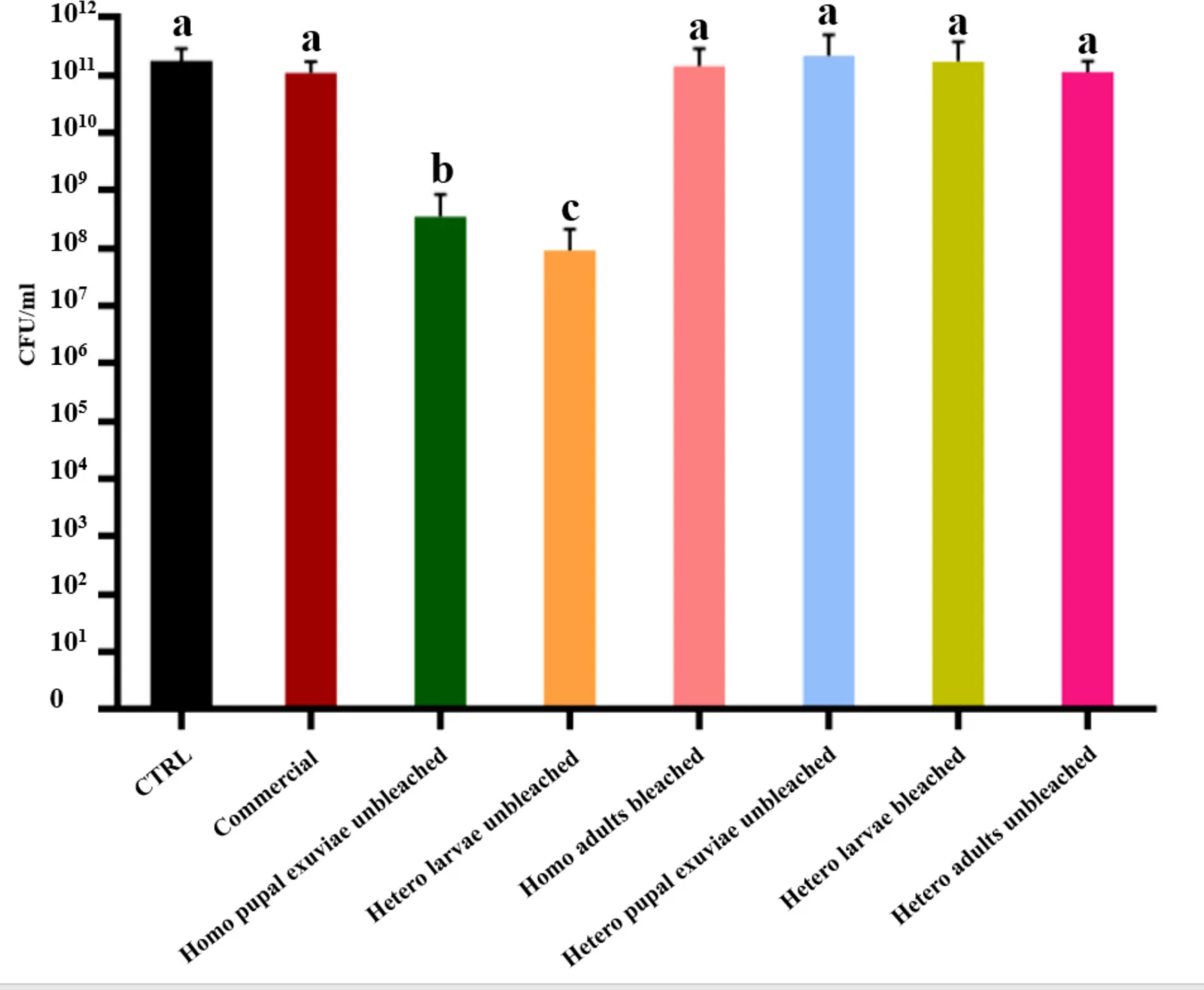
Results of CFUs counting of bleached and unbleached chitosan that gave MIC statistically significant values, both homogeneously and heterogeneously deacetylated, from larvae, pupal exuviae and adults of *H. illucens* at 0.5 mg/mL concentration on *S. epidermidis*. The black bar indicates the bacterial culture control, while the red bar represents commercial chitosan result. Different letters indicate significant differences (*p* < 0.05) between CFUs/mLof the bacterial culture alone and that of bacteria treated with each treatment. Data are analyzed with one-way ANOVA and Bonferroni *post hoc* test

These results were confirmed by CFUs counting (Fig. 6), in which only homogeneous unbleached chitosan from pupal exuviae and heterogeneous chitosan from unbleached larvae revealed a decrease of CFUs/mL. Indeed, these two samples yielded a colony count of 1.3 × 10⁸ and 3.3 × 10⁷ CFUs/mL, respectively, demonstrating a slight bactericidal activity with a reduction of three and four orders of magnitude compared to control.

**Fig. 7:**
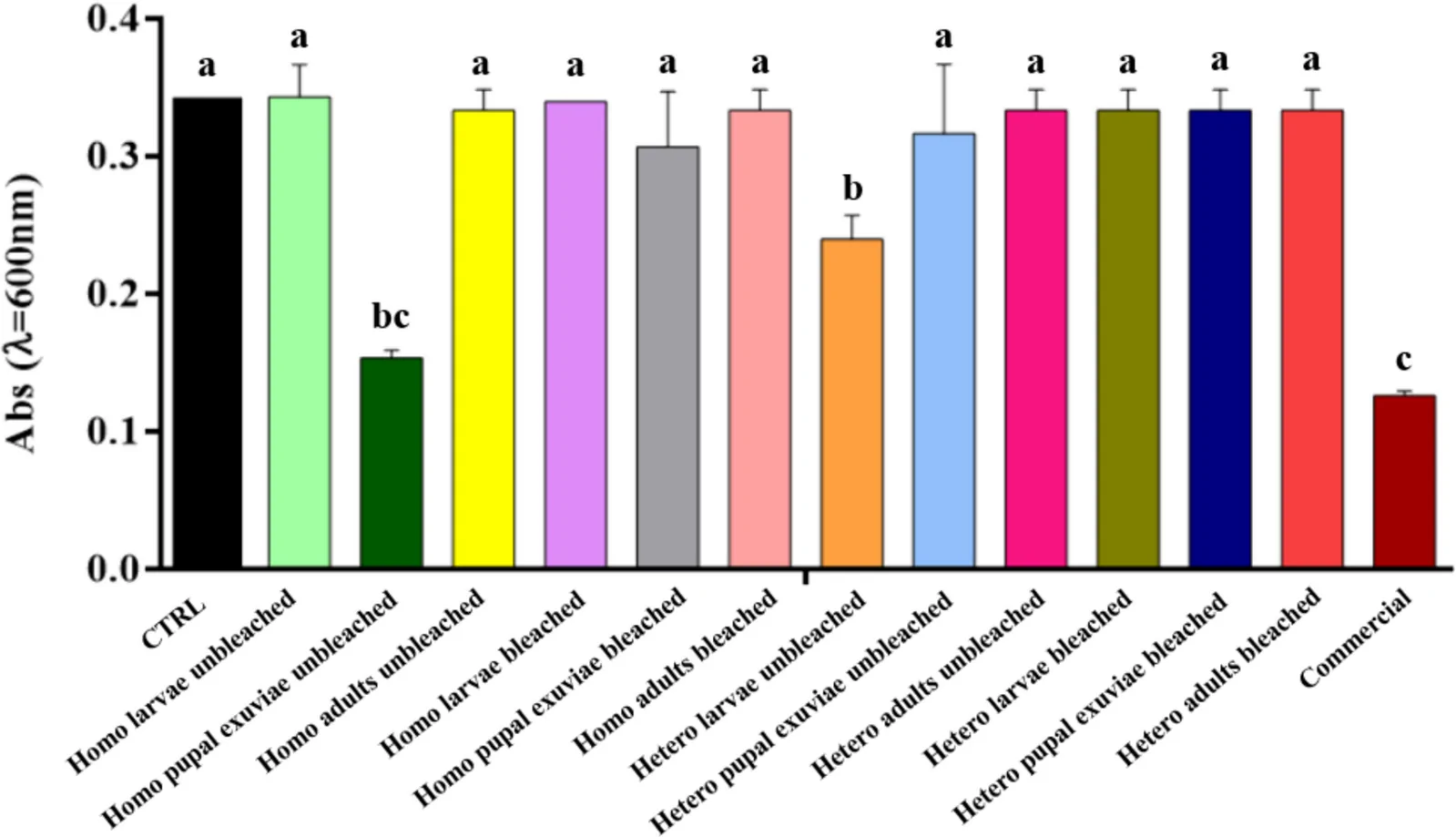
Results of microdilution assay of bleached and unbleached chitosan, both homogeneously and heterogeneously deacetylated, from larvae, pupal exuviae and adults of *H. illucens* at 0.5 mg/mL concentration on *S. agalactiae*. The black bar indicates the bacterial culture control, while the red bar represents the commercial chitosan result. Different letters indicate significant differences (*p* < 0.05) between absorbance values of the bacterial culture alone and those of bacteria treated with each treatment. Data are analyzed with one-way ANOVA and Bonferroni *post hoc *test

Treatment of chitosan samples from *H. illucens* on *S. agalactiae* showed high absorbance values (Fig. 7). Specifically, no significant differences were found between the samples obtained with the different deacetylation methods (heterogeneous and homogeneous), nor between those subjected to the bleaching step. This finding suggests that, in specific cases of the culture tested, these treatments did not have a significant impact on this bacterial species.

The only treatments that showed a significant reduction were those with the heterogeneous chitosan from unbleached larvae and those with the biopolymer obtained from homogeneously deacetylated unbleached pupal exuviae. This sample proved to be the most effective of the insect samples and showed no significant difference compared to chitosan from crustaceans, which resulted in the greatest reduction in absorbance.

**Fig. 8:**
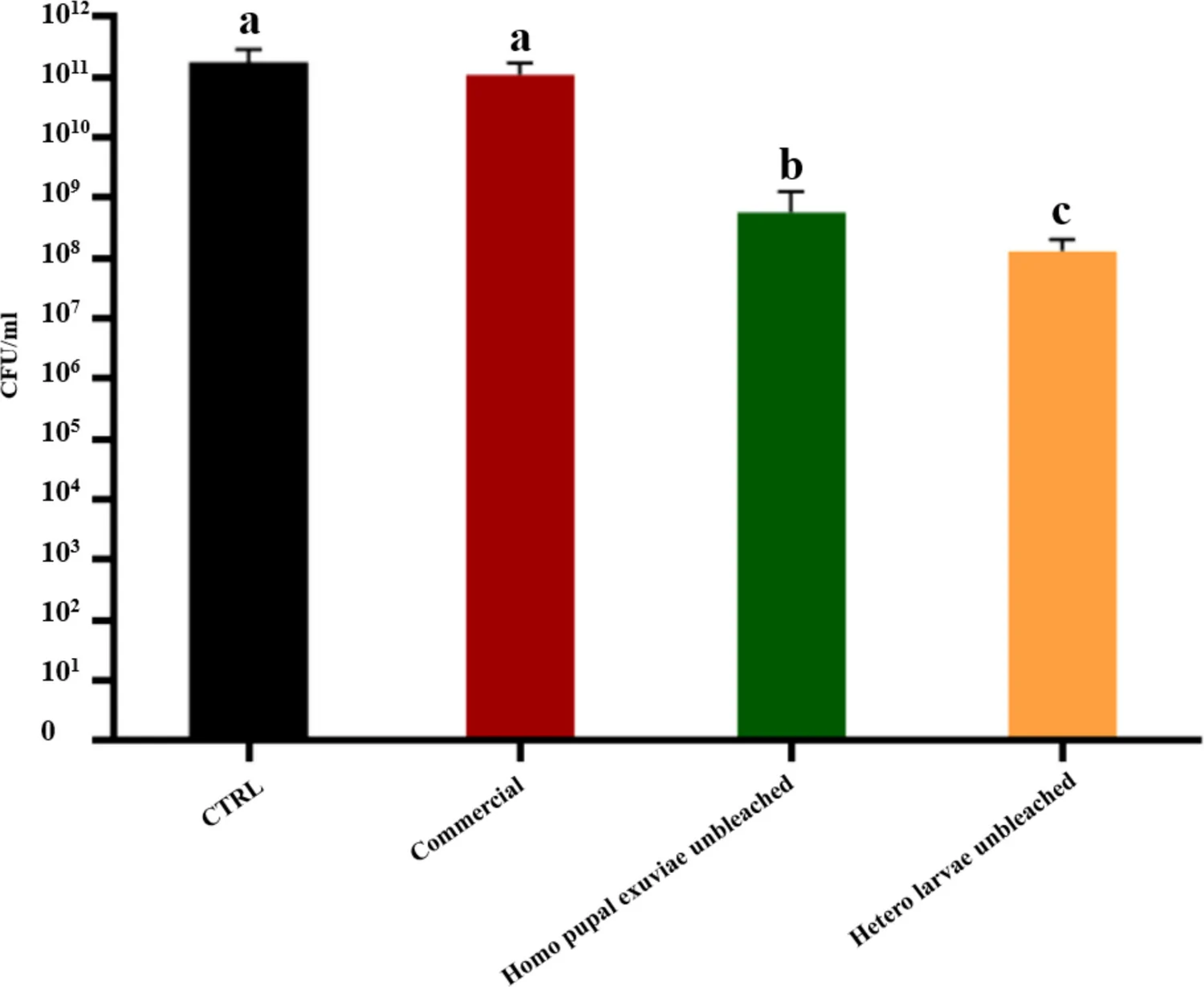
Results of CFUs counting of bleached and unbleached chitosan that gave MIC statistically significant values, both homogeneously and heterogeneously deacetylated, from larvae, pupal exuviae and adults of *H. illucens* at a 0.5 mg/mL concentration on *S. agalactiae*. The black bar indicates the bacterial culture control, while the red bar represents the commercial chitosan result. Different letters indicate significant differences (*p* < 0.05) between CFUs/mL of the bacterial culture alone and those of bacteria treated with each treatment. Data are analyzed with one-way ANOVA and Bonferroni *post hoc* test

The bactericidal effect on *S. agalactiae* has been tested only with the samples for which statistical differences in absorbance reduction were found, specifically homogeneous unbleached chitosan from *H. illucens* pupal exuviae and heterogeneous unbleached chitosan from larvae (Fig. 8). These samples gave a colony count value of 3.3 × 10⁸ and 1.2 × 10⁸ CFUs/mL, respectively. Results were statistically significant compared to control and commercial chitosan.

As previously detected on *E. faecalis*, the bactericidal activity of the samples was not strong enough to be considered bactericidal, but the antimicrobial effect could be defined more as a bacteriostatic activity.

## Discussion

Works in the literature had demonstrated the effectiveness of chitin, the non-deacetylated form from which chitosan is derived, in inducing the expression of the *HBD-2* peptide, but in the form of nanofibrillar systems (Azimi et al. 2020, 2024; Coltelli et al. 2022). Unlike chitin in fibrillar form, which has mainly a structural and strengthening function in biomaterials, chitosan can directly modulate the cellular immune response and can be employed in different pharmaceutical formulations, making it highly versatile in terms of application. The present findings confirm and expand these observations, showing that insect-derived chitosan can act as a potent stimulator of *HBD-2* expression in keratinocytes. The fact that both heterogeneous and homogeneous preparations were able to induce significant upregulation indicates that the immunomodulatory activity is a consistent feature of *H. illucens* chitosan, regardless of the specific preparation method. Further investigation will be useful in order to identify the specific molecular features responsible for insect biopolymer induction. These results found evidence also in insect and plant models, in which chitosan administration has been shown to enhance the expression levels of defensin and abaecin in honeybees (Saltykova et al. 2010a, 2010b) and of defensins 1, 2, and 3 in peanut hairy root culture (Pankaew et al. 2023).

Concerning direct antimicrobial activity, our findings confirmed that *H. illucens*-derived chitosan can exert a measurable antimicrobial effect against multiple bacterial strains, although primarily in a bacteriostatic rather than bactericidal way. This aligns with existing literature suggesting that the mechanism of action of chitosan relies on electrostatic interactions between the positively charged amino groups of chitosan and the negatively charged bacterial cell wall, leading to increased membrane permeability and growth inhibition rather than immediate cell death (Guarnieri et al. 2022). In some works, the commercial biopolymer allowed for lower inhibitory values than those identified for insect chitosan in this study, but using chitosan encapsulated in nanostructures. For example, *Lactobacillus acidophilus* chitosan nanoparticles gave a MIC value of 10 μg/mL (El-Mongy et al. 2023), while biopolymer nanoparticles, in the study by Pandey et al. 2024, gave MIC and MBC values of 0.31 mg/mL. Conversely, Jose et al. (2022) also studied the effect of lemon extract-mediated chitosan nanoparticles on *E. faecalis*, obtaining a MIC at 62.5 mg/mL and an MBC of 250 mg/mL, confirming that the active concentration at which we observed a significant growth reduction in our analysis was a better result, even when considering the treatment of the bacterial culture with chitosan alone. Concerning the antimicrobial activity on *S. epidermidis,* the growth reduction values of 0.5 mg/mL observed in our study are comparable to the results obtained by Amato et al. 2021. Indeed, in that work, the MIC value reported for chitosan alone was 0.5 mg/mL, while functionalising the biopolymer with other molecules this value could also be lower. On the contrary, other studies conducted on chitosan from squid pens (*Doryteuthis* spp.) detected MIC and MBC values that were lower than the growth reduction values obtained from our research, both testing the biopolymer alone and in nanoparticle systems (Marangon et al. 2020). As previously observed for *E. faecalis*, the bactericidal activity of the samples against *S. agalactiae* was weaker and could be better characterized as bacteriostatic rather than bactericidal. It is well recognised in the literature that chitosan oligosaccharides (COS) exhibit synergistic antimicrobial activity with antibiotics against *S. agalactiae*, group B streptococcus (GBS) (Asadpoor et al. 2021). Yildirim-Aksoy et al. (2019) demonstrated the inhibitory activity of chitosan at concentrations four times higher than ours (2 mg/mL vs. 0.5 mg/mL). According to their theory, chitosan at concentrations ≥ 0.2% exhibits significant antibacterial activity against *S. agalactiae*, whereas at lower concentrations, chitosan does not inhibit growth; indeed, it may stimulate the proliferation of *S. agalactiae*, probably because it is used as a carbon source. However, our work showed that even at lower concentrations, chitosan has bacteriostatic, rather than bactericidal, activity.

In general, the better results obtained with unbleached samples may be attributed to their higher molecular weight, a key factor influencing antimicrobial activity. Indeed, in the literature, the correlation between the molecular weight and the antimicrobial activity of the biopolymer is still lacking (Guarnieri et al. 2022). However, based on the results obtained, it can be hypothesised that samples that do not undergo the bleaching process, having longer polymer chains act according to the mechanism whereby they create a barrier around the bacterial cell wall that prevents nutrients from entering and thus causes microorganism death (Zheng and Zhu 2003).

Given the increasing need for biopolymers with both antimicrobial and immunomodulatory properties, insect chitosan could represent an innovative material for use in wound healing formulations, topical treatments, or drug delivery systems. The observation that direct antibacterial activity was only evident at the highest concentration tested suggests that the primary mechanism of action is indirect, mediated by the stimulation of host defense peptides such as *HBD-2*, rather than through direct bactericidal activity helping to reduce the risk of resistance development compared to classical antibiotics.

## Conclusions

*H. illucens*-derived chitosan, obtained from larvae, pupal exuviae, and adult insect biomass through both heterogeneous and homogeneous deacetylation, demonstrated antimicrobial properties against clinically relevant Gram-positive pathogens, namely *E. faecalis*, *S. epidermidis*, and *S. agalactiae*.

However, the observed activity at 0.5 mg/mL was primarily bacteriostatic, with microbial growth inhibition comparable to, or in some cases, greater than commercial crustacean chitosan.

Significantly, *H. illucens* chitosan was also shown to stimulate the expression of the *HBD-2* gene in keratinocytes, confirming its indirect antimicrobial and immunomodulatory properties.

This dual mechanism, specifically direct inhibition of bacterial growth and enhancement of host innate immune response, underscores the biopolymer potential against antimicrobial-resistant pathogens. In this perspective, the use of *H. illucens* as a chitosan alternative source represents an ecologically sustainable and economically viable alternative to the conventional crustacean-based source that can be efficiently employed in pharmaceutical and biomedical fields.

## Data Availability

The datasets used and/or analysed during the current study are available from the corresponding author on reasonable request.
